# Identification and Analysis of Sex-Biased Copy Number Alterations

**DOI:** 10.34133/hds.0121

**Published:** 2024-03-11

**Authors:** Chenhao Zhang, Yang Yang, Qinghua Cui, Dongyu Zhao, Chunmei Cui

**Affiliations:** ^1^ Department of Biomedical Informatics, Center for Noncoding RNA Medicine, State Key Laboratory of Vascular Homeostasis and Remodeling, School of Basic Medical Sciences, Peking University, 38 Xueyuan Rd, Beijing 100191, China.; ^2^ Department of Biochemistry and Biophysics, School of Basic Medical Sciences, Peking University Health Science Center, Beijing 100191, China.; ^3^ School of Sports Medicine, Wuhan Institute of Physical Education, No.461 Luoyu Rd. Wuchang District, Wuhan 430079, Hubei Province, China.

## Abstract

**Background**: Sex difference has long been recognized at cancer incidence, outcomes, and responses to therapy. Analyzing the somatic mutation profiles of large-scale cancer samples between the sexes have revealed several potential drivers of cancer with sex difference. However, it is still a demand for in-depth scrutinizing the sex-biased characteristics of genome instability to link the clinical differences for individual cancer type. **Methods**: Here, we utilized a published framework devised to specifically compare the copy number profiles between 2 groups to identify the sex-biased copy number alterations (CNAs) across 16 cancer types from the The Cancer Genome Atlas Program database, and dissected the impact of those CNAs. **Results**: Totally, 81 male-biased CNA regions and 23 female-biased CNA regions in 16 cancer types were found. Functional annotation analysis showed that several critical biological functions associated with sex-biased CNAs are shared in multiple cancer types, including immune-related pathways and regulation of cellular signaling. Most sex-biased CNAs have a substantial effect on transcriptional consequence, where the average of over 68% of genes have a linear relationship with CNAs across cancer types, and 14% of those genes are affected by the combination of the sex and copy number. Furthermore, 29 sex-biased CNA regions show latent capacity to be sex-specific prognostic biomarker such as CNA on 11q13.4 for head and neck cancer and lung cancer. **Conclusions**: This analysis offers new insights into the role of sex in cancer etiology and prognosis through a detailed characterization of sex differences in genome instability of diverse cancers.

## Introduction

Sex differences in cancer incidence, prognosis, mortality, and therapeutic responses have been extensively reported [1,2]. Most nonreproductive cancers exhibit a higher occurrence rate in men, except for thyroid cancer and gallbladder cancer [3,4]. Across the majority of cancer types, male patients show widely elevated mortality rates such as more than 4 times mortality rate found in bladder cancer and poor prognosis compared to female patients [5,6]. Notable sex disparities are also observed in the treatment of immune checkpoint inhibitors. For instance, anti-programmed cell death-1 therapy resulted in a higher overall survival and better response rates in female individuals with non-small-cell lung cancer compared to male patients [6]. Conversely, Unger et al. revealed a higher risk of severe symptomatic adverse events in women receiving chemotherapy, immunotherapy, or targeted therapies than those of men based on the data of 202 clinical trials [7]. The reasons for these sex disparities are partially attributed to the differences in exposure to established risk factors and expected protection by sex hormones for women [6]. On the other hand, the genetic and molecular biases contributing to these observations are equally critical, including diverse variations, gene expression, and DNA methylation [8–10]. Understanding these biases will contribute to the development of sex-specific clinical therapeutics.

The accumulation of genomic alteration events drives the development of cancers. Copy number alteration (CNA) is characterized by the aberration of a larger fraction of genome than other genetic variations and is widespread in cancer [11]. Moreover, CNAs have been demonstrated to tightly associate with the cancer development, diagnosis, and prognosis. For example, the increased incidence of amplification of *MYC*, *YAP1*, and *MMP13* is associated with the occurrence of brain metastases in lung cancer [12]; Killcoyne et al. [13] presented that the genomic copy number features can contribute to the early detection of esophageal cancer. Given that the role of CNAs in cancer and the ongoing genomic instability driving tumor heterogeneity, identifying the differential CNAs between males and females can offer novel insight into the mechanism underlying sex disparity in cancer outcomes beyond doubt.

Previous studies have documented sex differences in the frequency and density of somatic mutations between male and female cancer patients by using The Cancer Genome Atlas Program (TCGA) and Pan-Cancer Analysis of Whole Genomes (PCAWG) databases [8–10]. Nevertheless, only limited functional variation events are found, and it still needs in-depth investigation of linking this molecular difference to the cancer outcomes between the sexes. In this work, through a strict pipeline on comparing the copy number profiles of male and female patients presented by [14], we identified the CNA regions showing sex difference in 16 cancer types using the TCGA database. Further analysis of these sex-biased CNAs unveils crucial biological processes potentially linked to distinct cancer outcomes between the sexes and sex-related prognostic biomarkers in specific cancer types. Our results provide valuable insights into the role of sex in the etiology, diagnosis, and precision medicine of cancer.

## Materials and Methods

### Data collection and processing

Copy number profiles for the TCGA dataset were acquired through TCGAbiolinks (version 2.25.3) of R package (version 4.3.1) [15], including bladder urothelial carcinoma (BLCA), colon adenocarcinoma (COAD), glioblastoma multiforme, head and neck squamous cell carcinoma (HNSC), kidney renal clear cell carcinoma (KIRC), kidney renal papillary cell carcinoma (KIRP), brain lower grade glioma (LGG), liver hepatocellular carcinoma (LIHC), lung adenocarcinoma (LUAD), lung squamous cell carcinoma (LUSC), pancreatic adenocarcinoma (PAAD), pheochromocytoma and paraganglioma (PCPG), rectum adenocarcinoma (READ), sarcoma (SARC), stomach adenocarcinoma (STAD), and thymoma (THYM). Copy number profiles of Memorial Sloan Kettering (MSK) were obtained from cBioPortal (https://www.cbioportal.org/) and only primary cancer samples were selected [16,17]. Sample information for TCGA and MSK were shown in Tables S2 and S5, respectively. The gene expression profiles and clinical information for TCGA cohort were downloaded from UCSC Xena (https://xenabrowser.net/hub/). Genes with zero expression across more than 80% of samples were removed. This study used the TCGA database, which is publicly available. GISTIC2.0 was executed individually for male and female cohorts in each cancer [18]. The reference genome “hg38.UCSC.add_miR.160920.refgene.mat” was chosen, and other parameters were set with default values. All analyses excluded X and Y chromosomes.

### Analysis of genome instability

The percentage of CNA genes occurring gain or loss in genome was defined as PGCG and was calculated by the output matrix of GISTIC2.0, which has mapped segments into corresponding genes according to their genome positions. First, genes with a score of 0 in the matrix were considered to be normal copy number, genes with a score of 1 or 2 were considered to occur copy number amplifications, and genes with a score of −1 or −2 were considered to occur copy number deletions. We then calculated the percentage of genes with the CNA across chromosome arms for each sample. The aneuploidy score and loss of heterozygosity for each patient were obtained separately from previous studies [19,20]. Wilcoxon’s test was used to test the differences of genomic instability indicators between the sexes. To control the false discovery rate (FDR < 0.05), the method of Benjamini and Hochberg was adopted to adjust *P* values in the R program.

### Identification of sex-biased CNA regions

We used the case-control copy-number analysis using Gaussian process latent difference (CNGPLD) method to identify the genome regions with differential copy number aberrations between male and female patients [14]. CNGPLD is a tool that uses a statistical framework (Gaussian process latent difference model) to capture covariance structure of copy number data between 2 groups and achieves controlling the FDR at the region level [14]. Here, we applied this approach to compare 2 copy number data distribution of male and female cohorts for identifying genomic regions with different CNAs between sexes. The threshold of FDR < 0.05 and absolute value of latent difference (Ldiff) > 0.1 were used to define the sex-biased CNA region. CNAs in a genomic region exhibit a higher degree of changes in the male cohort compared to the female cohort when Ldiff is greater than 0, and vice versa. We obtained human oncogenes from ONGene database (http://www.ongene.bioinfo-minzhao.org/) and human tumor suppressor genes from TSGene database (https://bioinfo.uth.edu/TSGene/) to investigate whether the cancer-related genes are located at these sex-biased CNA regions [21,22].

### Functional analysis of sex-biased CNA regions

To explore the biological functions potentially influenced by sex-biased CNAs, a weighted gene functional enrichment analysis based on hypergeometric test, called WEAT, was employed (https://www.cuilab.cn/weat/) [23]. Based on the fact that the essentiality of genes varies for an organism, WEAT introduces a prior weight of gene into enrichment analysis and enables to reveal several critical pathways omitted by conventional functional enrichment analysis methods. First, we obtained the genes located on those sex-biased CNA regions, named as sex-biased CNA genes. Then we selected gene importance calculator score as the gene weights and the gene ontology terms to perform functional analysis. The threshold for significantly enriched terms was set at FDR < 0.05.

### Transcriptional and clinical relevance of sex-biased CNA regions

To test the effect of sex-biased CNA regions on gene expression, we used multiple linear regression to model the relationship of mRNA abundance with sex, copy number, and interaction of copy number and sex. The gene copy number data is derived from the output file of GISTIC2.0. And the mRNA abundance is represented by the normalized expression value with log-transformed FPKM. And the *P* value threshold of 0.05 was used.

For the clinical relevance of sex-biased CNA regions, we first obtained the clinical information of all samples. For any sex-biased CNA regions, we subsequently divided either the female or male cohort into 2 groups according to samples harboring current CNA or not. To be specific, we executed survival analysis on each gene of one sex-biased CNA region, and the most significant result represented the effect of this region. The relevance of survival outcomes with each sex-biased CNA were tested by log rank test. The significantly differential survival outcomes exclusively observed in either female or male were considered as the sex-specific prognostic biomarker. The R package survival (v3.5-5, https://CRAN.R-project.org/package=survival) was used to perform survival analysis. And the *P* value threshold of 0.05 was used.

## Results

### Overview of sex biases in genome instability across cancers

**Fig. 1.**
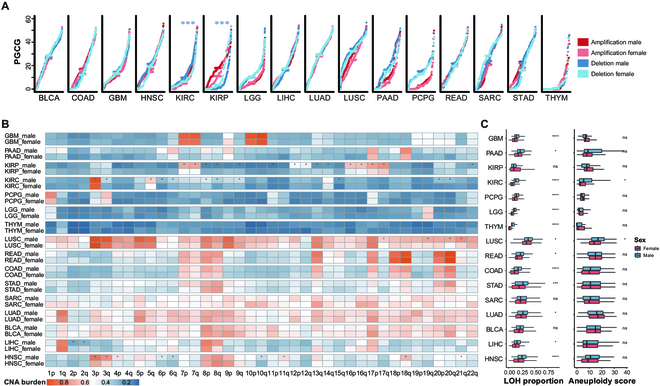
Genome instability features between males and females. (A) The scatter plots depicting the percentage of genes with CNAs in the genome are displayed for each type of cancer, categorized by amplification (in red) and deletion (in blue). The horizontal lines represent median values. Significance labels are incorporated with the adjusted *P* threshold of 0.05 (Wilcoxon's test, * FDR < 0.05, ** FDR < 0.01, *** FDR < 0.001, **** FDR < 0.0001). (B) Heatmap represents the average proportion of CNAs on chromosome arms in male (top) and female (bottom) patient cohorts. (Wilcoxon's test, * FDR < 0.05). (C) The comparison of 2 genome instability indicators (LOH proportion and aneuploidy score) between males (blue boxes) and females (red boxes) (Wilcoxon's test, * FDR < 0.05, ** FDR < 0.01, *** FDR < 0.001, **** FDR < 0.0001).

To explore sex disparities in cancers, we first deleted tumour samples lacking sex information and retained only cancer types with the number of both male and female samples exceeding 50 in the TCGA database, resulting in a cohort of 5947 tumour samples (3658 males, 2289 females) covering 16 cancer types (Tables S1 and S2). Our analysis focused on sex differences in autosomal genome instability, thus those of sex chromosomes were excluded. We first compared the number of DNA segments showing copy number aberration between the sexes in each cancer type. Significant sex difference was only observed in head and neck cancer (HNSC) with a FDR of 6.98 × 10^-6^ (Wilcoxon’s test, Fig. S1A). Then we found that the number of genes with CNAs varies among difference cancer types as described in Fig. S1B. Thus, we investigated the presence of sex bias in the coverage of CNAs at the gene level by the percentage of genes with CNAs in genome (PGCG). As shown in Fig. 1A, a majority of cancers exhibit a comparable distribution of PGCG between the sexes except for several cancer subtypes. Especially, Male patients displaying higher PGCG with deletion than females were observed in KIRP (FDR = 5.73 × 10^-4^, Wilcoxon’s test) and KIRC (FDR = 2.58 × 10^-4^, Wilcoxon’s test). Subsequently, we interrogated the CNA burden across 22 autosomes between the sexes. As shown in Fig. 1B, certain cancers have relatively low abundance of CNA burden on most chromosomes, particularly, thymoma, LGG and PCPG, while others such as lung cancer and BLCA show enriched CNAs across autosomes. Five out of 16 cancer types showed significant sex differences on several chromosome arms, including LIHC, HNSC, lung squamous cell cancer (LUSC), KIRC and KIRP with FDR < 0.05 (Fig. 1B). Previous study reported that the amplification of chromosome 2p was associated with drug resistance in chronic lymphocytic leukemia [24]. A tendency of female patients with LIHC relative larger CNA burden on chromosome 2p compared with males can be found (FDR = 4.09 × 10^-3^, Wilcoxon’s test), which might lead to the distinct cancer outcomes between males and females. The results suggest that limited cancer types show sex disparity with regard to the density of CNA in either whole genome or different chromosomes. In addition, we observed that 2 cancer types (KIRC and LUSC) show consistent tendency with the CNA density in other genomic instability indicators including aneuploidy score from [20] and loss of heterozygosity (LOH) from [19], where genome instability tends to be more prevalent within the male patient cohort (Fig. 1C). These results indicate that sex differences in the genome instability remain obviously cancer-specific, notably in kidney, lung, liver, and head and neck cancers.

### Identification of sex-biased CNA regions

**Fig. 2.**
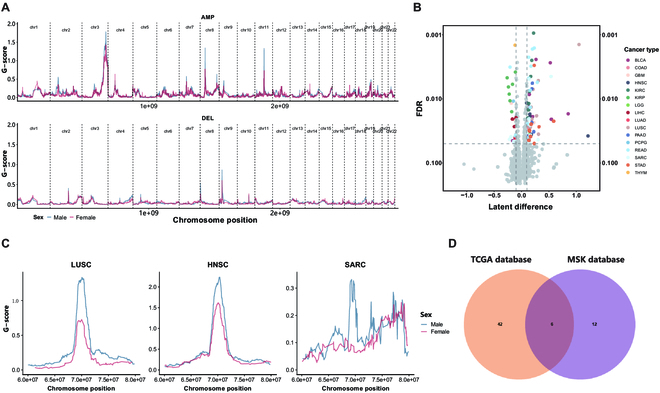
Identification of sex-biased CNA regions. (A) Genome-wide distribution of G-score for male and female LUSC patients. Blue lines represent male, red lines represent female. (B) The volcano plot represents all sex-biased CNA regions identified by the CNGPLD tool across 16 cancers. Different colors are selected to mark sex-biased regions in different cancers that reach the threshold (FDR < 0.05 and absolute value of latent difference > 0.1). (C) The distribution of G-scores for sex-biased CNA region on 11q13.2 were showed across 3 cancer types (LUSC, HNSC, and SARC). (D) The overlap of sex-biased CNA regions based on the data of the TCGA database and the MSK database.

Sex differences in genome instability emerges as a form of vast arrays of differential CNAs between males and females. The previous studies have detected the sex-biased CNAs in multiple cancers by applying conventional statistical method such as Fisher’s exact test to test each loci independently [9,10]. However, the CNAs of adjacent loci are highly linked, which can cause standard adjustment methods to fail in controlling the FDR [14]. To dissect the sex-biased CNAs in each cancer type, we used a published framework called CNGPLD that is specifically designed for comparative copy-number analysis such as discovering differentially amplified or deleted regions in metastatic cancer compared to primary cancer on the basis of a Gaussian process prior and is conductive to disclose more genuine differential CNAs [14]. Although the distribution of CNAs along genome are similar between the sexes, for example, Fig. 2A for lung cancer and others shown in Fig. S2, we collectively identified 104 significantly sex-biased CNA regions across 16 cancers (Fig. 2B and details seen in Table S3), with the majority of these regions being enriched in male samples (81 male-biased regions and 23 female-biased regions). The median length of these sex-biased CNA regions is 3.9Mb (4.0Mb for male-biased CNA regions and 2.7Mb for female-biased CNA regions). In addition, 81% of sex-biased CNA regions show amplificated copy number. Among these cancer types, READ exhibits the most number of sex-biased CNA regions with 14, whereas only one sex-biased CNA region in PCPG and thymoma are found (Fig. 2B). The fewer number of sex-biased CNA regions in LGG, thymoma, and PCPG may stem from the overall lower abundance of CNA burden across chromosomes. Meanwhile, there are several sex-biased CNA regions are shared among multiple cancers. For example, the amplification on 11q13.2 shows sex disparity in HNSC, LUSC, and Sarcoma (SARC). In the case of HNSC, we found 9 significant sex-biased regions with FDR < 0.05, with the CNA on 11q13.2 having the greatest difference (Ldiff = 1.21, FDR = 3.78 × 10^-2^, Fig. 2C) between the sexes. As well as LUSC and SARC, the amplification of this region also significantly enriches in male patients compared to female patients (LUSC: Ldiff = 1.05, FDR = 1.42 × 10^-3^, SARC: Ldiff = 0.17, FDR = 3.66 × 10^-2^). Moreover, we found that this region harbors 4 oncogenes (Table S4) including *FGF4*, *CCND1*, *FGF3*, and *CTTN*, of which *CCND1* is a key regulator in cell cycle control and associated with malignant biological behavior in solid tumors and contributed to immunosuppression [25]. This result suggests a potentially shared mechanism that may contribute to sex differences in the incidence and drug response of these cancer types. Similarly, the significantly gained region on 17q12 in male-derived samples occur at various types of cancer containing COAD, HNSC, READ, and STAD (Fig. S3). In addition, it is observed that there are cancer-related genes in 84% sex-biased CNA regions (Table S4). Our analyses indicate that the sex-biased CNA regions occur in most cancers and that these regions contain either tumor suppressor genes or oncogenes contributing to the differences in cancer development between the sexes.

To validate the reliability of determined sex-biased CNA regions, the same pipeline was deployed on the copy number data stored on Memorial Sloan Cancer Center (MSK), which is based on the targeted sequencing technology [16]. We only focused on 7 cancer subtypes with more than 200 cancer samples, and a total of 7215 samples were acquired (Table S5). There are 18 CNA regions showing sex difference in bladder urothelial carcinoma, LUSC, PAAD, READ, and glioblastoma (Table S6). Notably, 6 shared regions between sex-biased CNAs from TCGA and those from MSK are found, accounting for 33.33% of all sex-biased CNA regions from MSK (Fig. 2D). For example, in lung cancer, the amplification on 11q13.2 exhibits sex disparity in both datasets, which partially suggests the robustness of CNGPLD framework and the potential of digging out true positive loci with differential CNAs.

### Functional enrichment analysis of sex-biased CNA genes

**Fig. 3.**
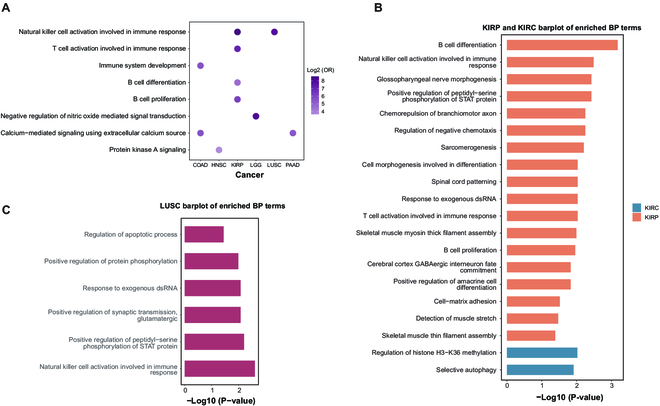
Functional enrichment analysis of sex-biased CNA genes. (A) The enriched functions (FDR < 0.05) of sex-biased CNA genes shared between multiple cancer types (COAD, HNSC, LGG, LUSC, and PAAD). (B) The enriched biological processes (BP) of sex-biased CNA genes in 2 kidney cancers (KIRC and KIRP), only the significant terms are shown (FDR < 0.05). (C) The enriched biological processes (BP) of sex-biased genes CNA in LUSC (FDR < 0.05).

To further discern the biological pathways affected by CNAs exhibiting sex disparity, we extracted the genes located on these sex-biased CNA regions to perform functional enrichment analysis through WEAT [23], which is a tool for weighted enrichment analysis considering that the effects of different genes are distinct even though they involve in one pathway. As depicted in Fig. 3A and Table S7, there are some biological processes shared in different cancer types, including regulation of signal transduction and immune-related pathways. Particularly, genes on sex-biased CNA regions in head and neck cancer are enriched in protein kinase A signaling; those genes of pancreatic cancer and COAD are associated with calcium-mediated signaling using extracellular calcium source; genes of LGG are enriched in negative regulation of nitric oxide mediated signal transduction. Similarly, multiple immune-related pathways are associated with the sex-biased CNA regions in multiple cancer types, including natural killer cell activation in kidney papillary cell cancer and lung squamous cell cancer, immune system development in COAD, and B cell proliferation and differentiation and T cell activation in kidney papillary cell cancer. In addition, we observed several specific biological processes associated with sex-biased CNA genes. For 2 renal cell cancer subtypes, there are separately male-biased CNA regions dominance in KIRP and female-biased CNAs dominance in KIRC, and thus completely distinct pathways linked to sex-biased CNAs are found (Fig. 3B). In KIRC, the genes on sex-biased CNA regions are enriched in histone methylation regulation and selective autophagy, while those genes of KIRP are involved in immune regulation and multiple cell differentiation processes. Previous studies also reported a marked sex disparity in kidney papillary carcinoma in the processes related to immunity [26]. Other than the biological processes accordant with that of KIRP, sex-biased CNA genes of lung squamous cell cancer are also related to protein phosphorylation and apoptotic process (Fig. 3C). These results can assist in tracing the origin of sex disparities in cancer etiology and outcomes.

### Transcriptional and clinical relevance of sex-biased CNA regions

**Fig. 4.**
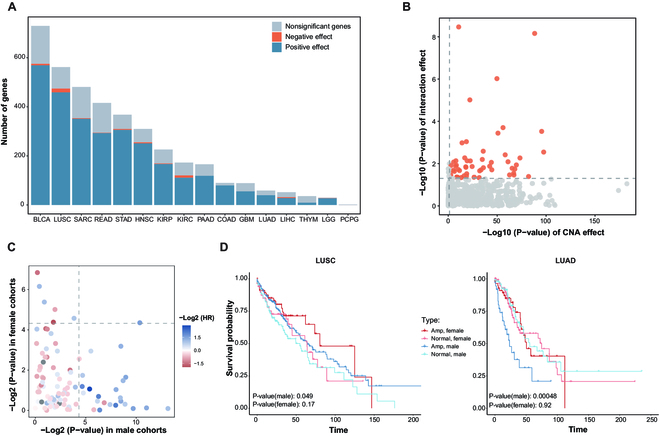
The effect of sex-biased CNA regions on transcription and prognosis. (A) The bar plot shows the number of sex-biased CNA genes whose transcription is affected by CNA across 16 cancers. The blue bar represents a positive correlation between copy number and expression level, and the orange bar represents a negative correlation. (B) The effect of CNA and sex on the mRNA abundance of sex-biased CNA genes for LUSC. Genes with significant interaction terms of the sex and copy number are highlighted with orange points (*P* < 0.05). (C) The effect of sex-biased CNA regions on the overall survival of male and female cohorts, respectively, and the points locating at the top left and bottom right regions are the potential sex-specific prognosis markers. The points are colored according to the log2 of the HR. Only the more significant HRs between male and female cohorts were selected to represent this region's HR. (D) The prognosis effect of the sex-biased CNA region on 22q11.22 are significant difference (*P* < 0.05) between the sexes in 2 lung cancers (LUAD and LUSC). The lines in dark and light red represent female patients with amplification and without CNAs, respectively. The lines in dark and light blue represent male patients with amplification and without CNAs, respectively.

The gene expression is intuitively influenced by the aberrant gains or losses of the DNA segment. Previous studies have revealed the existence of vast genes with sex disparity in their expression [27,28] and a substantially positive relationship between CNAs and the gene expression [29]. Therefore, the multiple linear regression model was employed to ascertain the effect of the sex-biased CNA regions on the transcriptional consequence of corresponding genes. The model fit the mRNA abundance with the sex of individual, copy number and the interaction between the sex and copy number. An average 68% of genes present significantly disrupted expression due to CNAs across 16 cancer types (Fig. 4A and Table S8). Among them, the mRNA abundance of 93% of the sex-biased CNAs genes in LGG are affected by their copy number, while thymoma and PCPG have an extremely low proportion in gene expression influenced by the CNA, 27% (10 out of 36 genes) and 0% (0 out of 2 genes), respectively. There is a significantly positive linear relationship between alterations in gene copy number and their expression for the majority of genes (Fig. 4A). For instance, 97% of genes demonstrate a positive effect on their expression due to the CNAs in lung squamous cell cancer, and 99% in bladder cancer. Furthermore, there are a number of genes whose abundance is collectively affected by both sex and copy number, averaging 14% of sex-biased CNA genes across 16 cancer types (Table S9). As illustrated in Fig. 4B, 55 genes display a significant interaction (*P* < 0.05) of the sex and copy number in lung squamous cell cancer, where we found 7 well-established cancer-related genes including *CTTN*, *CDKN2B*, and *PTPN6*. The abundance of *PTPN6* is significantly affected by the sex of individual and copy number, where a significant positive correlation (*P* < 0.05) between copy number and mRNA abundance in males and no correlation in females are found (Fig. S4)*.* In head and neck cancer, there exist 37 genes such as *PPP1R1B* and *CDC6* with significant association between mRNA abundance and the interaction of the sex and CNAs. In terms of COAD, the combined effect of the sex and CNAs impacts 18 genes, accounting for 21% of sex-biased CNA genes (Table S9). Taken together, sex-biased CNAs have an observable potential to alter the transcriptome, especially for cancer-related genes, thereby further contributing to phenotypic difference of cancers between males and females.

Chromosomal aberrations are closely associated with the prognosis of cancer patients [30,31]. Correspondingly, we supposed that the impact of CNA regions with sex bias would be reflected in the divergent survival outcomes of male patients and female patients. To validate this, for a sex-biased CNA region, we separately compared the overall survival of patients with or without CNAs in male cohorts and female cohorts. As shown in Fig. 4C and Table S9, 29 out of 104 sex-biased regions display difference in the prognosis affected by CNAs between the sexes. To be specific, a sex-biased CNA region on 22q11.22 shows the opposite hazard ratio (HR) in male patients with amplification of this region for 2 lung cancer subtypes (LUAD: *P* = 4.76 × 10^-4^, HR = 0.38; LUSC: *P* = 4.87 × 10^-2^, HR = 1.40) (Fig. 4D), whereas there is no correlation between the CNA in this region and prognosis among female patients. The shared sex-biased CNA region on 11q13.4 between LUSC and head and neck cancer shows both sex-specific association with survival outcome (male: *P* = 0.874, female: *P* = 8.73 × 10^-3^ for LUSC; male: *P* = 1.12 × 10^-4^, female: *P* = 0.942 for HNSC, Fig. S5A). 1p/19q chromosomal codeletion has been recognized as the prognostic marker in glioma [32]. We noticed that solely male patients derived from LGG with the amplification of 1q32.1 present a poor prognosis (male: *P* = 2.42 × 10^-3^, HR = 0.45, female: *P* = 0.364, HR = 0.71, Fig. S5B). This result suggests the potential of several sex-specific markers associated with cancer prognosis in the sex-biased CNAs.

## Discussion

In this study, we conducted a comprehensive comparison of genomic instability between the sexes across cancers. We employed a tool specifically designed for the comparison of CNA profiles between 2 groups and identified 104 sex-biased CNA regions in 16 cancer types, where some shared sex-biased CNAs were observed between different cancer types. Functional enrichment analysis revealed that sex-biased CNA genes are involved in diverse cellular signaling and multiple immune-related pathways. Moreover, more than 60% of genes on these regions are significantly influenced by the CNAs, and approximately 14% of whose mRNA abundance are jointly regulated by the sex and the CNA. Survival analysis found that multiple sex-biased CNAs have the potential to be sex-specific prognostic biomarkers including 11q13.4 for LUSC and head and neck cancer, 1q32.1 for LGG. In conclusion, the identification of sex differences in the characteristics of genomic instability carries new insights for cancer biology research and personalized medicine.

To validate our results, we deployed the same pipeline on the MSK dataset to identify sex-biased regions. Notably, the sex-biased CNA regions exhibit a degree of overlap between MSK and TCGA indicating the robustness of the method of identifying the differential CNAs between the sexes. However, the data of MSK are derived from target-sequencing analysis focused on specific key cancer genes [33], which is not able to provide a comprehensive representation of the entire genome landscape. Moreover, the patient cohorts in MSK were heavily treated with advanced cancer [16], and another study also demonstrated the transformation of the genome landscape in metastatic treated stages compared to primary untreated tumor [34]. In future, additional comprehensive datasets are required to further validate the CNAs with sex difference.

Actually, the sex-associated bias in somatic variations across cancer types have raised concerns a few years ago [9,10], in which several driver genes with sex disparity at mutation level were found. We compared the results of our study with that of Li et al. and found a limited overlap in the genes with sex-biased CNAs in all cancer types, such as LUSC shared genes in the sex-biased regions 17q24.2. The method used in our study addresses the concern that the conventional statistical test fails to control the FDR due to the highly correlated copy number states of adjacent regions, and accordingly, the number of false positives of the identified sex-biased CNAs based on the CNGPLD method are relatively low. We also used Fisher’s exact test to identify sex-biased CNA genes with the threshold of P-values less than 0.05 in 7 shared cancers between the TCGA and MSK databases. Then only 9% of sex-biased CNA genes are shared between these 2 databases, while we revealed 33% overlapped CNA regions with sex disparity based on the CNGPLD method. This result further validates the robustness of the CNGPLD method. In addition to the widely reported sex differences in lung and kidney cancers previously, we also discovered multiple insightful sex-biased CNA regions exhibits clinical relevance for head and neck cancer and LGG. In general, our results can be a supplement to sex-biased genome characteristics.

Recently, multiple copy number signatures describing the mutational processes have been presented. Steele et al. [19] revealed no association between copy number signatures and the sex. However, we analyzed the sex difference in the 17 copy number signatures characterizing specific types of chromosomal instability [35] and found several cancer types with differential signatures between the sexes (Fig. S6). For example, male patients of COAD exhibit a higher frequency in CX5 associated with impaired homologous recombination; the occurrence of CX13, attributed to replication stress, is more prevalent in men than women with LUAD. These results partially documented the etiology difference of cancers between the sexes.

## Data Availability

The data that support the findings of this study are available from the TCGA database website and cBioPortal. Other analysis results are available in the supplementary materials.
