# Supplementary figures and images for: Identification and Analysis of Sex-Biased Copy Number Alterations
Source: Health Data Sci. 2024 Mar 11;4:0121. doi: 10.34133/hds.0121 (PMC11249066; doi:10.34133/hds.0121)

**A**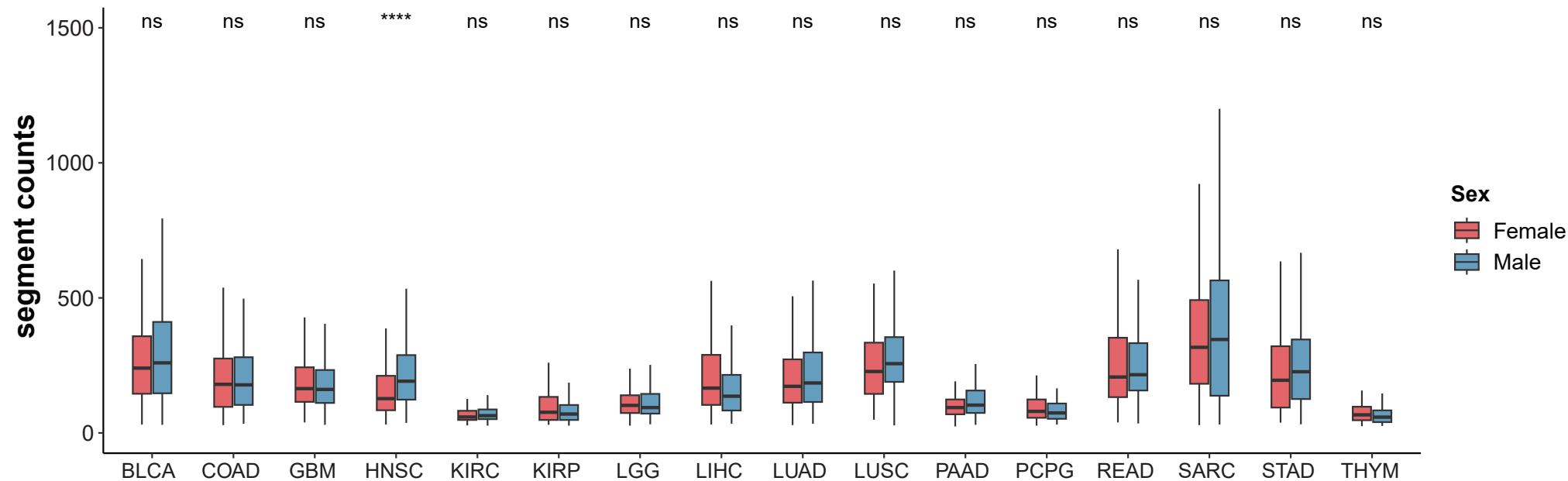**B**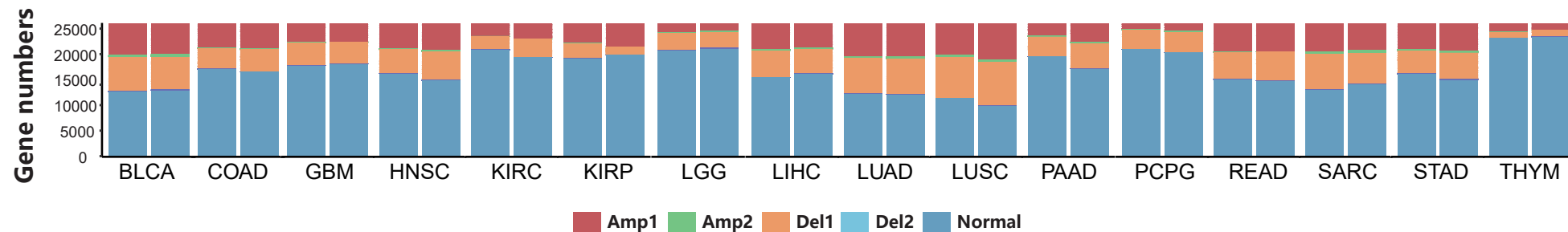

Supplement: Supplementary 1 — Figs. S1 to S6 Tables S1 to S10 [file hds.0121.f1.zip › FigS1.pdf]

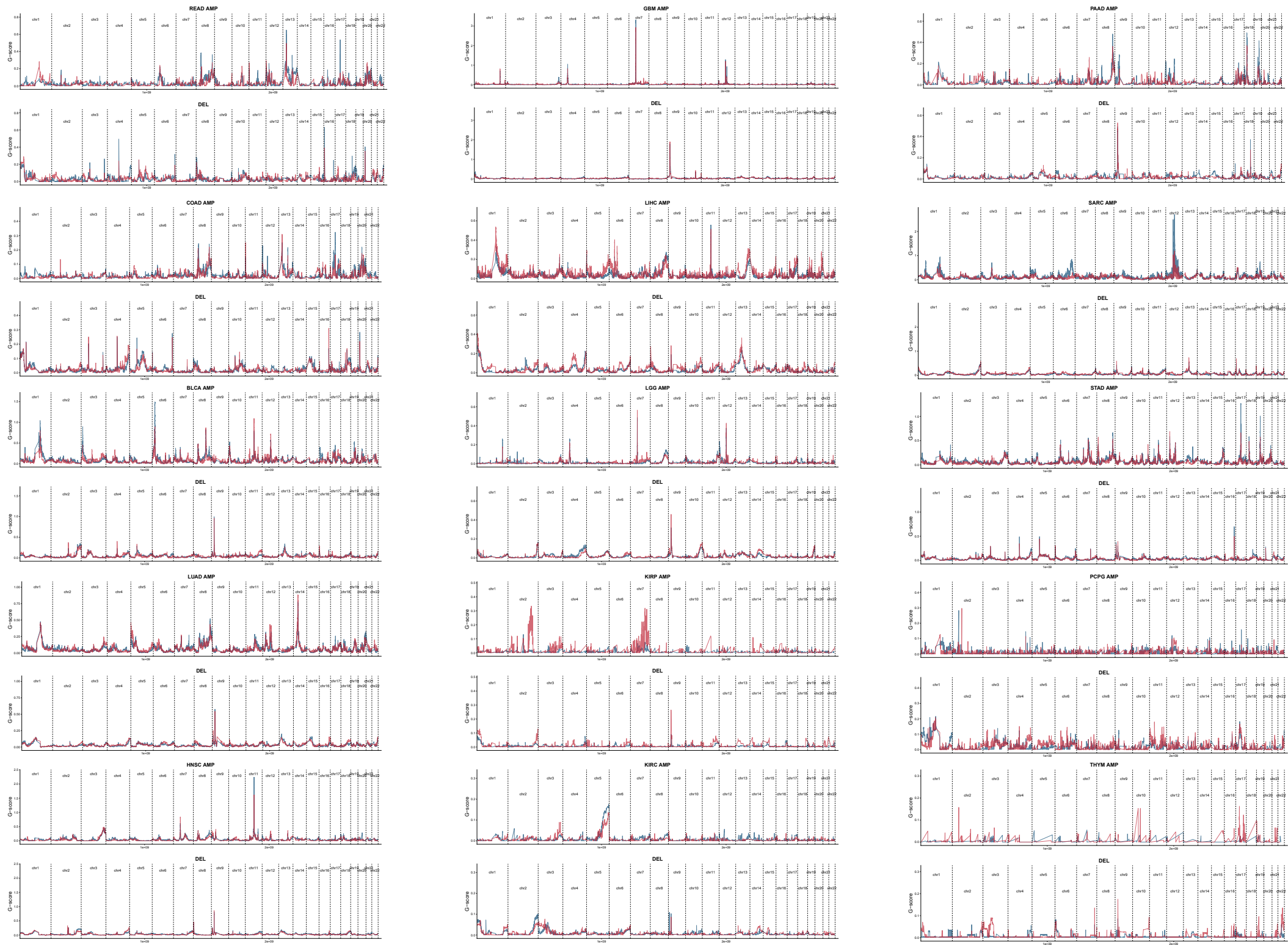

Supplement: Supplementary 1 — Figs. S1 to S6 Tables S1 to S10 [file hds.0121.f1.zip › FigS2.pdf]

**A**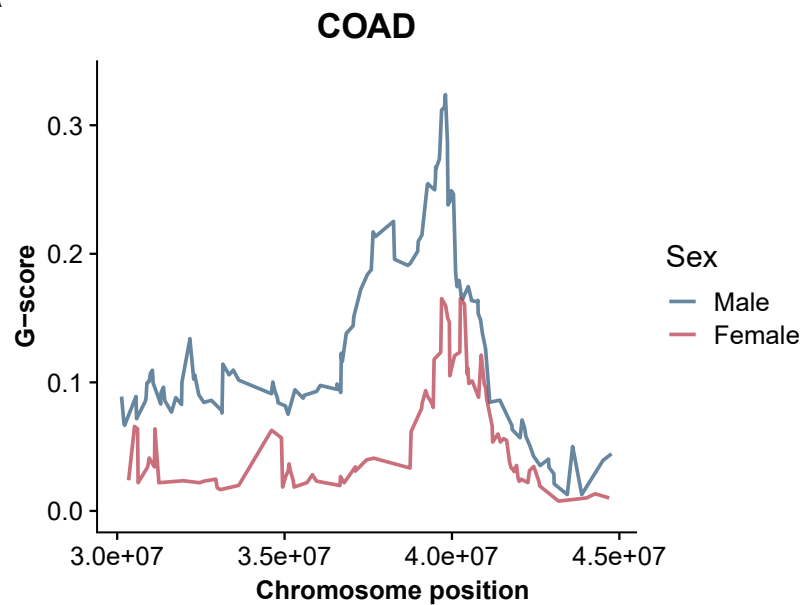**B**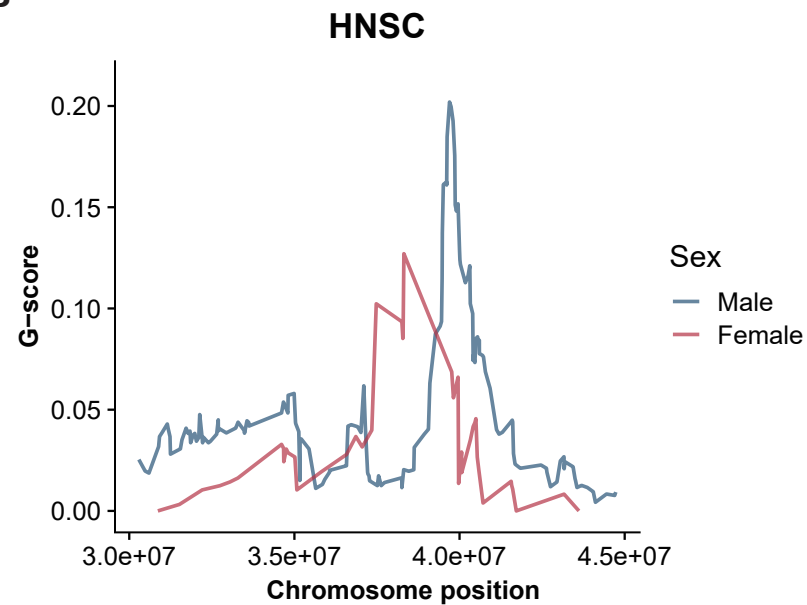**C**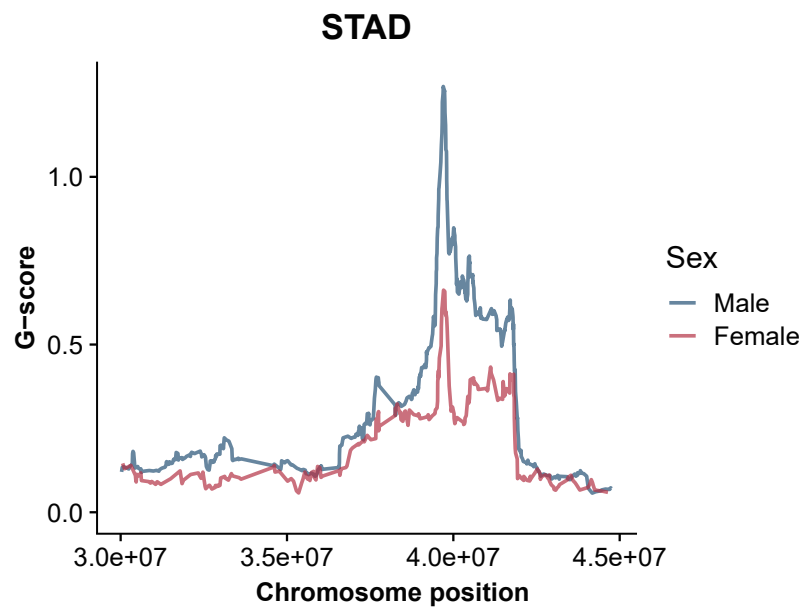**D**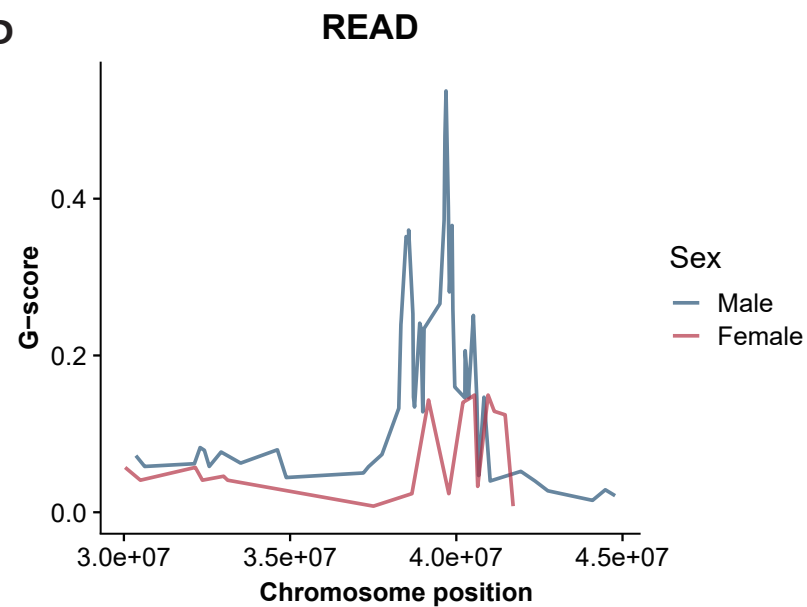

Supplement: Supplementary 1 — Figs. S1 to S6 Tables S1 to S10 [file hds.0121.f1.zip › FigS3.pdf]

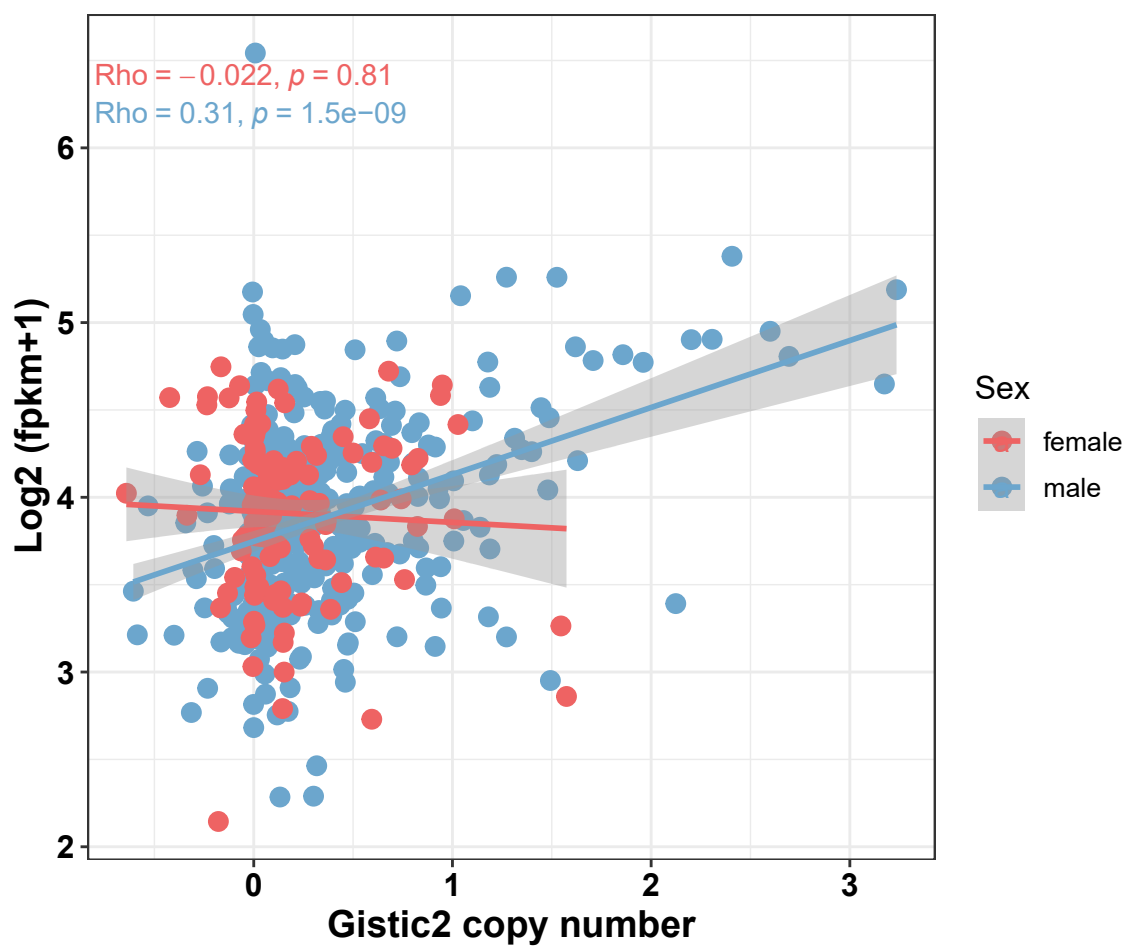

Supplement: Supplementary 1 — Figs. S1 to S6 Tables S1 to S10 [file hds.0121.f1.zip › FigS4.pdf]

**A**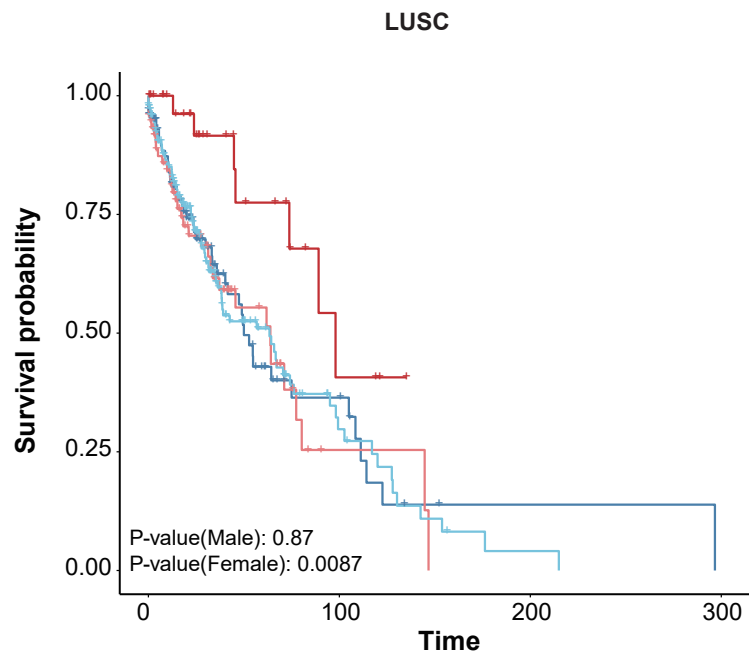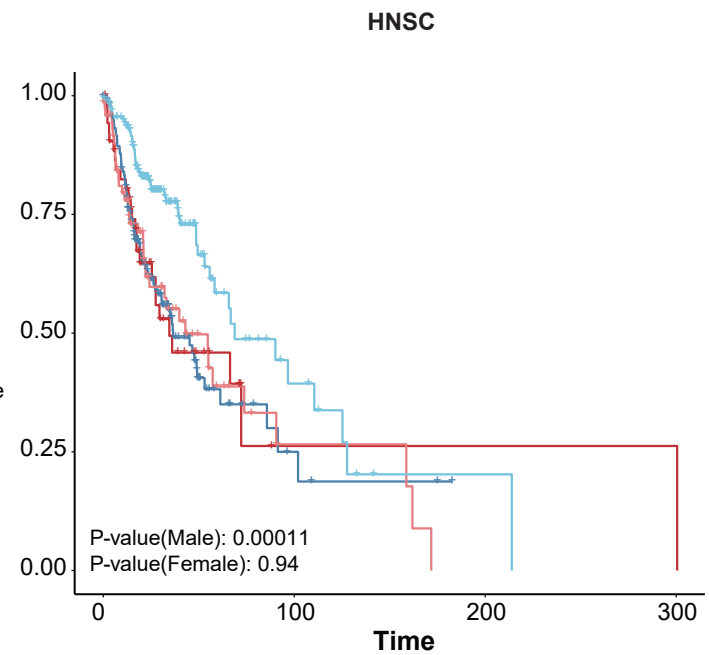**B**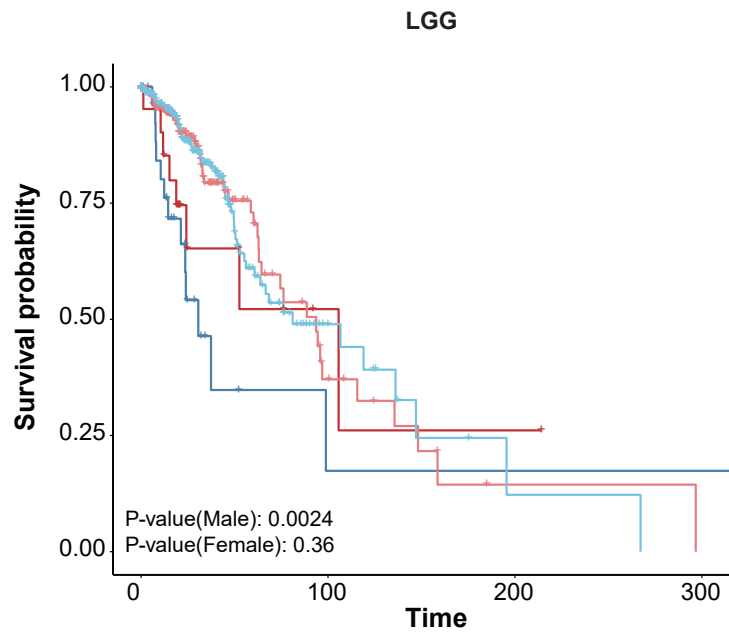

Supplement: Supplementary 1 — Figs. S1 to S6 Tables S1 to S10 [file hds.0121.f1.zip › FigS5.pdf]

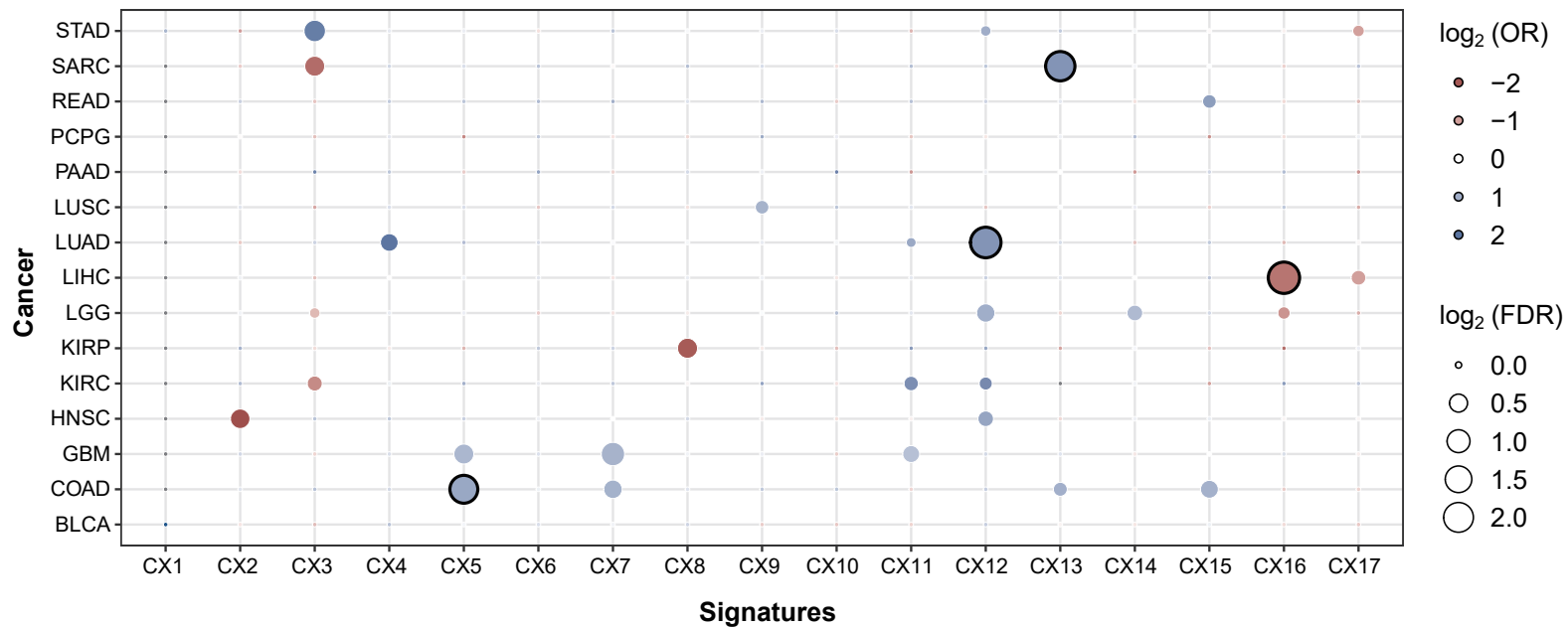

Supplement: Supplementary 1 — Figs. S1 to S6 Tables S1 to S10 [file hds.0121.f1.zip › FigS6.pdf]
